# Psychometric Properties of the Bermond–Vorst Alexithymia Questionnaire (BVAQ) in the General Population and a Clinical Population

**DOI:** 10.3389/fpsyt.2018.00111

**Published:** 2018-04-23

**Authors:** Lars de Vroege, Wilco H. M. Emons, Klaas Sijtsma, Christina M. van der Feltz-Cornelis

**Affiliations:** ^1^Department Tranzo, Tilburg School of Social and Behavioral Sciences, Tilburg University, Tilburg, Netherlands; ^2^Clinical Centre of Excellence for Body, Mind, and Health, GGz Breburg, Tilburg, Netherlands; ^3^Department of Methodology and Statistics, Tilburg School of Social and Behavioral Sciences, Tilburg University, Tilburg, Netherlands

**Keywords:** psychometric properties, alexithymia, Bermond–Vorst Alexithymia Questionnaire, validation study, somatic symptom and related disorders

## Abstract

**Introduction:**

The Bermond–Vorst Alexithymia Questionnaire (BVAQ) has been validated in student samples and small clinical samples, but not in the general population; thus, representative general-population norms are lacking.

**Aim:**

We examined the factor structure of the BVAQ in Longitudinal Internet Studies for the Social Sciences panel data from the Dutch general population (*N* = 974).

**Results:**

Factor analyses revealed a first-order five-factor model and a second-order two-factor model. However, in the second-order model, the factor interpreted as analyzing ability loaded on both the affective factor and the cognitive factor. Further analyses showed that the first-order test scores are more reliable than the second-order test scores. External and construct validity were addressed by comparing BVAQ scores with a clinical sample of patients suffering from somatic symptom and related disorder (SSRD) (*N* = 235). BVAQ scores differed significantly between the general population and patients suffering from SSRD, suggesting acceptable construct validity. Age was positively associated with alexithymia. Males showed higher levels of alexithymia.

**Discussion:**

The BVAQ is a reliable alternative measure for measuring alexithymia.

## Introduction

Sifneos (1) introduced the terminology of alexithymia to describe emotional deficiencies in patients suffering from classic psychosomatic disorders and epilepsy (2, 3). These patients were unaware of their feelings and their unawareness was accompanied by an inability to fantasize about their inner thoughts, feelings, and attitudes. Several studies report that alexithymia is a relatively stable trait rather than a state-dependent phenomenon (4–6). Alexithymia has been linked to neurobiological and neuropsychological characteristics such as the functioning of the “visceral” or “limbic” brain [e.g., MacLean (3)]. Furthermore, alexithymia has been associated with somatization (7, 8), somatoform disorder (9), and psychosomatic symptoms (10), and is considered a risk factor for the development of major depression (11), schizophrenia (12), psychosis (13), and eating disorders (14). Moreover, emotional deficiencies were found to have a negative impact on one’s health and were a potential obstacle for successful psychological treatment (15). Recently, De Berardis et al. (16) evaluated the relationship between alexithymia and suicide risk and recommended the assessment of alexithymia in clinical practice. This renders alexithymia important in research on understanding the onset and progress of medically unexplained symptoms and to further improve the effectiveness of psychotherapeutic interventions.

The conceptualization of alexithymia is ongoing, and several questionnaires have been developed to assess alexithymia: the two most frequently used questionnaires are the Bermond–Vorst Alexithymia Questionnaire (BVAQ) (17) and the twenty-item Toronto Alexithymia Scale (TAS-20) (18). Both questionnaires are self-report measures and both have good reliability (19). The TAS-20 operationalizes alexithymia as a constellation of three cognitive factors: difficulty identifying feelings, difficulty describing feelings, and external-oriented thinking (18). However, the TAS does not cover fantasizing, which Bagby et al. (20) and Bermond et al. (19) conceived as another essential feature of alexithymia. The absence of fantasizing motivated Bagby and colleagues to develop the Toronto Structured Interview for Alexithymia (TSIA) (21), which also measures fantasizing. The third factor of the TAS-20 and the TSIA (the externally oriented thinking factor) actually reflects how people have cognitions about their feelings to guide their behaviors, and so, it describes a possible connection between cognition and emotions, as is reflected in confirmatory factor analyses of the original TSIA (21). Another study of the TSIA (22) showed a four-factor model with difficulty identifying feelings and difficulty describing feelings item-facet sets nested under one higher-order factor labeled affect awareness, and the externally oriented thinking and the imaginal processes item-facet sets nested under a second higher-order factor labeled operatory thinking, consistent with the conceptualization of alexithymia as involving different domains in emotional processing and emotional experience.

The BVAQ takes the emotional aspects of alexithymia into account in a more explicit way. It uses a more comprehensive definition of alexithymia by operationalizing alexithymia as a constellation of five basic factors: ability to fantasize and fantasize about virtual matters (fantasizing), ability to identify emotions (identifying), looking for an explanation of emotional reactions (analyzing), ability to describe and/or communicate about emotional reactions (verbalizing), and ability to be emotionally aroused (emotionalizing). The inclusion of emotionalizing as a distinctive factor is a difference between the BVAQ and the TSIA and the TAS-20.

According to Vorst and Bermond (17), emotionalizing refers to the degree of emotional arousal by emotion-inducing events. However, considering emotionalizing as an aspect of alexithymia is subject to debate (23), because emotionalizing might not describe differences in awareness of feelings but rather differences in physiological arousal (20). The BVAQ enables the clinician to assess both cognitive and affective aspects of alexithymia in a more explicit way. Hence, the BVAQ provides clinicians with clinically relevant information.

### Internal Validity of the BVAQ

For justifiable use of the BVAQ, both in research and clinical settings, it is important that its psychometric properties are well understood. Although the factorial structure and the psychometric properties of the BVAQ have been the subject of several studies [e.g., Bagby et al. (20); Bekker et al. (24); Bermond et al. (25)], six potential issues necessitate further research: (a) indeterminacy of the BVAQ’s factor structure, (b) use of inadequate groups such as student samples, (c) use of small sample sizes, (d) invalid respondent answers due to lack of motivation to fill out the BVAQ, (e) lack of comparison of the BVAQ between groups with expected different alexithymia levels, and (f) factor structures for indicative and counter-indicative items.

Several studies replicated the first-order five-factor structure of the BVAQ, including the factors identifying, verbalizing, analyzing, fantasizing, and emotionalizing [e.g., Bagby et al. (20); Bekker et al. (24); Bermond et al. (25); Deborde et al. (26); Vorst and Bermond (17)], but Hornsveld and Kraaimaat (27) found poor fit. Bermond et al. (25) reduced the five factors to two second-order factors, representing a cognitive dimension and an affective dimension. These two second-order factors were obtained using principal component analysis (PCA) followed by both orthogonal (varimax) and oblique (oblimin) rotation and were corroborated by findings in neuropsychological research (28). Other studies were unable to replicate the second-order factors (20) or the affective dimension (24). Hence, our study aims at addressing internal validity by exploring the first-order and second-order factor structure of the BVAQ using exploratory factor analyses (EFA).

### External Validity

Different explanations may be given for the ambiguity in the first-order and the second-order factor structures, some of which pertain to the external validity of the BVAQ. Most studies used small clinical samples or student samples, usually psychology students, and may have played a role in ambiguous findings regarding factor structure so far. Student samples cannot be considered to adequately represent the populations of interest, such as the general population or clinical populations. Another problem is that PCA or EFA using small samples may be overly sensitive to sampling fluctuation (29), limiting the generalizability of the sample results to the population. Sample size limitations were rarely recognized in the literature. Hence, in this study, we used a large sample and we explored the external validity of the BVAQ in several ways.

### Ecological Validity

In this study, we used panel data from a large sample from the general population. A disadvantage of panel data is that respondents complete the questionnaire under artificial conditions because the outcomes of the BVAQ are not the respondent’s interest. As a result, respondents may not be motivated to complete the selected questionnaires (thus inducing selection bias), complete the questionnaire randomly, or tend to give only extreme responses (i.e., either 1 or 5 scores). This might result in data having questionable validity that provides a biased picture of the questionnaire’s ecological validity. Invalid data may also explain ambiguous factor-analysis results. Person-fit analysis (30) may signal traitedness for a limited number of respondents, thus casting doubt on the validity of their data (31).

### Construct Validity, Differences Between Populations

The BVAQ renders assessing differences between alexithymia scores obtained from different populations possible. Differences are likely to be found between the general population and patients suffering from somatic symptom and related disorder (SSRD) (32), which replaced the somatoform disorders (33). Somatoform disorders were related with alexithymia, and we expect that the same relationship exists for patients suffering from SSRD. Therefore, for investigating construct validity, medical patients suffering from a high expected likelihood to suffer from alexithymia were included in the study. We anticipated that these patients scored higher on alexithymia than non-patients. Previous studies suggest that alexithymia mediated effectiveness of psychotherapy (34). Patients were recruited from a specialty mental health outpatient clinic for patients suffering from SSRD. The data were collected during intake for treatment, hence patients might be more honest with respect to their possible alexithymia symptoms than people from the general population who were investigated without personal treatment objective. Observed mean differences in BVAQ scores between the general population and SSRD patients provide further evidence of the questionnaire’s construct validity.

### Construct Validity, Indicative and Counter-Indicative Items

Another validity issue with the BVAQ is the use of indicative and counter-indicative items. Questionnaires containing indicative and counter-indicative items, in the literature often referred to as balanced scales [e.g., Vigneau and Cormier (35)], may reveal additional factors related to response styles, or additional factors may arise because positively and negatively worded items might tap slightly different attributes, thus limiting construct validity. Subtle differences between subpopulations with respect to the interpretation of indicative and counter-indicative items might also explain differences between the factorial structures found in different BVAQ studies. Interpretation differences have received little attention so far. To further understand the possible wording effects and possible implications for using the BVAQ in clinical practice, we performed two EFAs, one for the indicative items (i.e., *I find it difficult to express my feelings verbally*) and one for the counter-indicative items (i.e., *I often use my imagination*).

### Scoring

Bermond–Vorst Alexithymia Questionnaire item scores may be added to obtain test scores for items loading on the first-order factors, the second-order factors, and for all the items in the questionnaire. In general, sum scores are more reliable when the number of items grows larger, but when additional items tap different traits, the conceptual interpretation of the scores may be less clear. For example, total BVAQ scores are most reliable, but equal scores might reflect different alexithymia profiles, thus hampering the clinical interpretation of total scores. Therefore, sum scores have to be based on subsets of items allowing a clear interpretation. Vorst and Bermond (17) advocated the use of second-order BVAQ scores because these scores preserve about 70% of the variance of the first-order scores and maintain a clear meaning. Researchers and clinicians may want to use first-order scores to investigate how different alexithymia aspects correlate with other variables, but then, the question arises whether first-order scores have additional value compared to second-order scores. Reise et al. (36) showed that, under certain conditions, total scores on a multi-factor questionnaire may provide more reliable information about specific trait aspects than scores based on single factors. We compared the psychometric properties of sum scores based on first-order factors and second-order factors, including sum-score reliability, and explored whether or not first-order test scores were more reliable than the second-order test scores.

Finally, we provided norms based on normative data from the general population to enhance the interpretation of individual BVAQ sum scores. Because, in former studies, results regarding gender and age differences were ambiguous (17, 37), we explored gender and age differences with respect to the BVAQ.

## Materials and Methods

### Participants

#### General Population Sample

Data were used from the Longitudinal Internet Studies for the Social Sciences (LISS) panel (www.lissdata.nl) collected by CentERdata (Tilburg University, The Netherlands). The LISS panel constitutes a representative panel that consists of 4,500 households, comprising 7,000 Dutch-speaking adults from the general population, permanently residing in the Netherlands, who participate in monthly internet surveys. The panel was drawn from the population register by Statistics Netherlands. Households without access to the internet were provided with a computer and an internet connection. Panel members complete online questionnaires for about 15–30 min on a monthly basis. Relevant ethical safeguards were met with respect to the participant’s confidentiality and consent. More detailed information about the LISS panel is provided in Scherpenzeel et al. (38).

**Figure 1:**
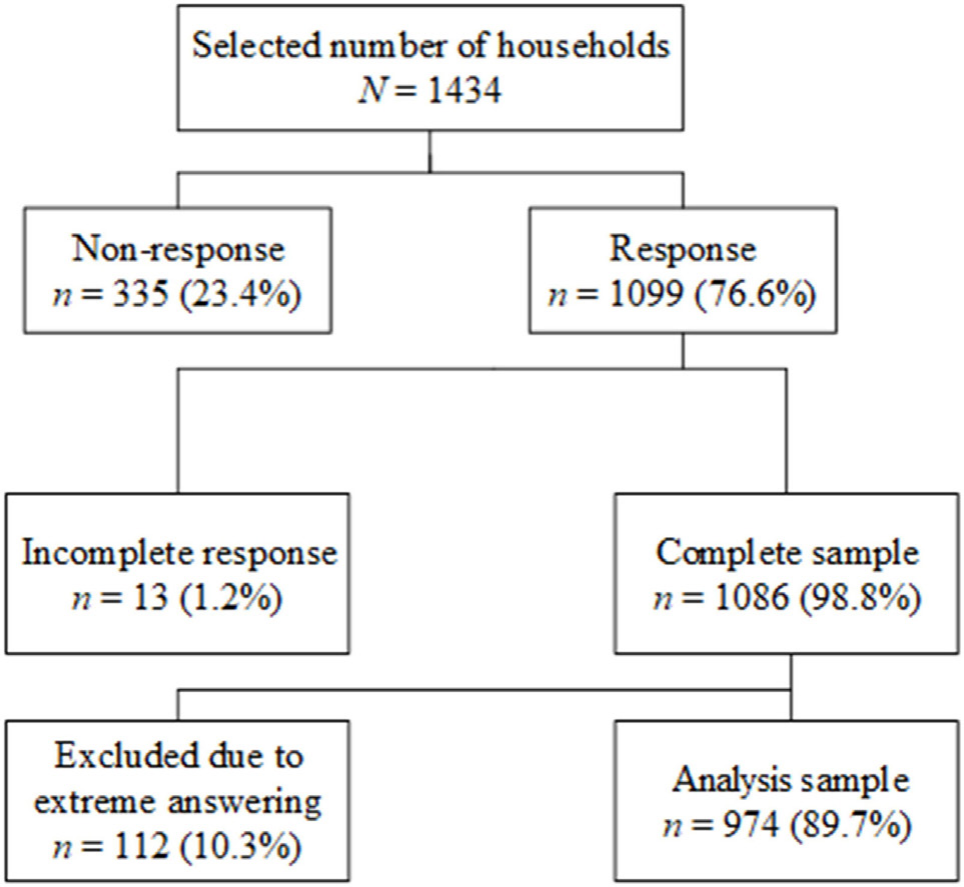
Overview of sample composition general population.

For this study, a random sample of 1,434 panel members from the LISS panel were invited by email to complete an online questionnaire that included the BVAQ, but 335 respondents (23.4%) did not respond. Thirteen participants (1.2%) started filling out the BVAQ, but did not complete the survey and were considered as non-responders. Hence 1,086 (98.8%) participants completed the questionnaire. Table 1 shows the sample characteristics of both responders (47% males and 53% females) and non-responders (44.8% males and 55.2% females). In the analysis sample, men were on average older than women [*t*(972) = − 2.95, *p* = 0.003, *d* = 0.19]. Responders were significantly older [mean difference = 12.3, *t*(1,432) = 12.72, *p* < 0.001, *d* = 0.78], better educated (*p* = 0.03, Cramer’s *V* = 0.10), and more often engaged in a relationship (*p* < 0.001, Cramer’s *V* = 0.20) than non-responders. Figure 1 shows how the final sample was obtained. The Dutch version of the BVAQ was digitalized and propounded to the LISS panel. After data collection, the raw data were transformed following the scoring syntax suggested by Vorst and Bermond (17).

**Table 1:** Sociodemographic characteristics of the study sample and the non-responders.

Characteristic	Study sample			Gender differences		Non-responders	Differences between study sample and non-responders	
				
	Total *N* = 974 M (SD)/*n* (%)	Men *n* = 458 M (SD)*/n* (%)	Women *n* = 516 M (SD)/*n* (%)	*p*	Effect size	*n* = 348 M (SD)/*n* (%)	*p*	Effect size
Gender							0.47	0.02^c^
Men	458 (47.0%)	–	–			156 (44.8%)		
Women	516 (53.0%)	–	–			192 (55.2%)		
Age	50.4 (17.2)	52.1 (17.4)	48.8 (16.9)	0.003	0.19^a^	37.7 (15.4)	<0.001	0.78^a^
Range	18–89	18–89	18–87			16–80		
Educational level*				0.09	0.10^b^		0.03	0.10^b^
Low (1–4)	284 (29.2%)	125 (27.3%)	159 (30.8%)			102 (29.3%)		
Medium (5)	211 (21.7%)	107 (23.4%)	104 (20.2%)			84 (24.1%)		
High (6–7)	479 (49.2%)	226 (49.4%)	253 (49.0%)			161(46.3%)		
Marital status				0.27	0.06^b^		<0.001	0.20^b^
Married	522 (53.6%)	254 (55.5%)	268 (51.9%)			133 (38.2%)		
Divorced	104 (10.7%)	46 (10.0%)	58 (11.2%)			29 (8.3%)		
Widow(er)	54 (5.5%)	19 (4.1%)	35 (6.8%)			7 (2.0%)		
Never	294 (30.2%)	139 (30.3%)	156 (30.0%)			179 (51.4%)		

*Based on the Verhage coding, which includes seven levels ranging from low (levels 1 through 4), medium (level 5), and high (levels 6–7) (39).

*^a^Cohen’s d*.

*^b^Cramer’s V*.

*^c^Phi*.

*Note: Non-responders did not complete the survey*.

#### Outpatient Clinic Sample

A sample of patients suffering from SSRD (*N* = 235) was used for external validation. All consecutive patients referred to the Clinical Centre of Excellence for Body, Mind, and Health (CLGG) situated in Tilburg, The Netherlands between August 2013 and April 2016 were included. The BVAQ was self-administered during the standard intake procedure. The Commission of Scientific Research of GGz Breburg approved to conduct this study (file number: CWO 2014-09). Patients gave consent to make use of their intake data for scientific research purposes. The inclusion criterion was age at least 18 years. Exclusion criteria were: patients were engaged in profession injury or personal procedures, were unable to come to CLGG, the primary focus was not physically related, a psychosis was present, ran active suicide risk (threatening), and were substance dependent.

### Instrument

#### Bermond–Vorst Alexithymia Questionnaire

Alexithymia was measured by means of the Dutch BVAQ. The BVAQ comprises 40 items; half of the items is alexithymia indicative and the other half is counter-indicative. Respondents rated their answer on a 5-point Likert scale ranging from “*this definitely applies*” to “*this in no way applies*.” All items were scored 1 through 5 such that higher scores reflect higher levels of alexithymia (17). The questionnaire comprises five subscales, which are identifying, verbalizing, analyzing, fantasizing, and emotionalizing, each in accordance with the five-factor model of alexithymia (17). Given item scores ranging from 1 to 5, the first-order test scores range from 8 to 40. Test scores on the cognitive factor were obtained by adding the total scores on the subscales identifying, analyzing, and verbalizing, meaning that test scores can range from 24 through 120. Test scores of the affective factor were obtained by adding the total scores on the subscales emotionalizing and fantasizing, thus producing test scores ranging from 16 through 80. Hence, high cognitive test scores represent problems with respect to the conscious experience of arousal accompanying emotions and high affective test scores reflect difficulties with respect to emotionalizing and fantasizing.

### Data Analysis

#### Internal Validity

Validity was investigated in a series of analyses. Because data were collected in low-stakes conditions, some respondents may not have been motivated to complete questionnaires seriously. Others may have used idiosyncratic response styles. Resulting aberrant item-response patterns were identified using person-fit analysis (30). Aberrant patterns were removed from the sample prior to EFA to obtain a sample without invalid item-response patterns. For person-fit analysis, we used the average normed number of Guttman errors (denoted *G_N_*) (40) across the subscales. Statistic *G_N_* can assume values between 0 (perfect fit) and 1 (extreme misfit). Following Emons et al. (41), we removed the highest 10% of the cases, which amounts to removing cases whose *G_N_* value was above 0.326. This cutoff is consistent with cutoffs suggested by Emons (40), based on simulations. This resulted in two (overlapping) samples, the complete sample and the analysis sample.

Exploratory factor analyses was done as follows. First, we used parallel analysis (42) in combination with minimum rank factor analysis (MRFA) [Ten Berge and Kiers (43), Timmerman and Lorenzo-Seva (44)] to determine the number of common factors. Like any factor-analysis approach, MRFA maximizes the item communalities given the number of factors (43), but MRFA does this such that the reduced correlation matrix is statistically correct. Therefore, MRFA allows valid estimates of the explained common variance (ECV) (45), which expresses the proportion of common variance explained by the hypothesized factors. Parallel analysis compares the percentage of variance explained by the factors with the percentage of variance explained by the same number of factors resulting from randomly generated data. In total, 500 random correlation matrices were generated by means of permutation of the raw data and subsequently analyzed by means of MRFA. Factors were considered meaningful if the percentage of variance these factors explained exceeded the percentage of variance the random-data factors explained. Because the BVAQ comprises ordinal items, showing both positive and negative skewness, some also showing excessive kurtosis, factor analysis of the polychoric correlation matrix was preferred (46). Parallel analysis was conducted by means of the free software program FACTOR version 10.3.01 (47).

Once the number of factors was determined, we investigated the factor structure using the configuration of the factor loadings. Promax rotation (48) was used to obtain the final rotated factor loadings. The presence of second-order factors was investigated by factor-analyzing the correlations between the first factors obtained using the first-order factor model. The final factor solution was again obtained using promax rotation. The final structures were inspected for adherence to a simple structure (48) and compared with the factorial structure Vorst and Bermond (17) found. EFAs were run in MPLUS7.1, using weighted least squares means and variance adjusted estimation (49) and R-package Psych (50).

Total-score reliability is commonly examined using coefficient alpha (51). The accompanying 95% confidence intervals (CIs) of coefficient alpha were obtained using the method of Feldt et al. (52) as implemented in the package cocron (53), which is also avalaible in R (54).

#### Construct and External Validity

To examine construct validity, we ran EFAs separately for the indicative and for the counter-indicative items. EFAs were run in MPLUS7.1 using weighted least squares means and variance adjusted estimation (49) and R-package Psych (50).

To examine external validity, we compared the BVAQ scores of the general population with the scores of SSRD patients to explore the degree to which the BVAQ discriminates between groups. Independent sample *t*-tests were done to compare the first-order and second-order BVAQ scores and Cohen’s *d* estimated effect size.

#### Scoring

To examine whether first-order test scores provided additional diagnostic information about the first-order factors that are more reliable than the information provided by the aggregated total scores, we used Haberman’s procedure (55). This procedure uses the proportional reduction in mean squared errors (PRMSE). The PRSME is conceptually similar to the reliability, and for first-order test scores, the PRMSE is equivalent to coefficient alpha. Large PRMSEs are desirable. PRMSEs were obtained using the R-package sirt (56).

Because of the expected differences between gender groups and age groups with respect to alexithymia, it might be useful to have separate norms for males and females and for different age groups. We first examined the relationship of gender and age with alexithymia to decide if separate norms for men and women and different age groups were needed. In case gender or age was associated with alexithymia, we used regression analysis to derive normative data [e.g., Oosterhuis et al. (57)]. This was done as follows. First, we regressed BVAQ scores on gender and age using a linear model with main effects only. The regression model provides estimates of mean BVAQ score as a function of gender and age. Second, for each respondent *i*, we computed a standardized residual; that is, *e_i_* = observed test score − expected test score, based on the estimated regression model. The distribution of the residuals served as normative reference distribution. The residuals were standardized using $e_{i}/S_{e_{i}}$ in which $S_{e_{i}}$ is the SD of the residuals. The standardized residual indicates the relative position of the individual’s score with respect to the mean in the population of persons having the same gender and the same age. To facilitate the interpretation of the standardized residuals, we converted standardized residuals to percentile values by means of the standard normal cumulative distribution. Model assumptions were tested by means of graphical inspection of the residuals. Analyses were done in Statistical Package for the Social Sciences for Windows version 22.0 (58).

## Results

Comparison of the background characteristics in the original sample and the analysis sample did not show any differences. Inspection of the misfitting cases showed unsystematic patterns. Six respondents scored “3” on all items, suggesting they did not seriously fill out the BVAQ. Consequently, they were considered as cases showing extreme response styles. The corresponding data records were removed from the sample, thus producing an analysis sample of 974 participants to be used for EFA.

### Factor Structure

Parallel analysis suggested five common factors. Model fit of the first-order five-factor model was acceptable (Comparative Fit Index = 0.94; Root Mean Square Error of Approximation = 0.046; Root Mean Square of the Residuals = 0.032). The first-order five-factor model explained 45.7% of the total variance and 68.3% of the common variance. Extracting a sixth factor only marginally improved the ECV to 71.7%, thus accounting for only a small proportion of common variance between the items. Therefore, we retained five first-order factors for further analysis.

Table 2 (columns 2–12) shows the standardized factor loadings for the first-order five-factor model and promax rotated factors, for the full sample and the analysis sample (only loadings above 0.3 are reported). In both samples, the loadings approximated a simple structure [e.g., Gorsuch (48)]; that is, for each factor, at least a few items only loaded predominantly on that specific factor. However, the pattern of loadings differed from the postulated five-factor structure (17), and results differed between the complete sample and the analysis sample. Based on the literature (17, 25), we initially labeled the factors as follows: verbalizing (F1), fantasizing (F2), identifying (F3), emotionalizing (F4), and analyzing (F5).

**Table 2:** Standardized factor loadings of the five-factor model for complete and analysis sample (i.e., without aberrant response patterns). Items are listed in clusters according to the subscales as suggested by Vorst and Bermond’s subscales. Only loadings of 0.3 or higher are reported.

	Complete sample					Analysis sample				
	F_1_	F_2_	F_3_	F_4_	F_5_	F_1_	F_2_	F_3_	F_4_	F_5_
**Items from Vorst and Bermond’s subscale verbalizing**										
i1	0.61				0.30	0.72				
i6	0.63					0.63				
i11	0.71					0.73				
i16	0.58					0.61				
i21	0.44				0.30	0.51				
i26	0.62					0.61				
i31	0.65		0.34			0.65				
i36	0.45					0.51				

**Items from Vorst and Bermond’s subscale fantasizing**										
i2		0.35			−0.34		0.40			
i7		0.67					0.70			
i12		0.77					0.77			
i17		0.56					0.57			
i22		0.87					0.89			
i27		0.77					0.77			
i32		0.64					0.67			
i37		0.51					0.58			

**Items from Vorst and Bermond’s subscale identifying**										
i3			0.55					0.48		
i8					0.59			0.58		
i13			0.59					0.54		0.34
i18					0.64			0.66		
i23					0.59			0.62		
i28			0.68					0.60		0.37
i33					0.68			0.65		
i38			0.64					0.62		

**Items from Vorst and Bermond’s subscale emotionalizing**										
i4				0.48					0.47	
i9				0.70					0.75	
i14				0.60					0.57	
i19				0.35	−0.49			−0.33	0.30	0.31
i24				0.51	0.39			0.31	0.51	
i29				0.59	−0.41				0.55	
i34				0.55					0.54	
i39				0.70					0.72	

**Items from Vorst and Bermond’s subscale analyzing**										
i5					0.36					
i10			0.40							0.41
i15										
i20			0.50							0.49
i25					0.38			0.39		
i30			0.45	0.30						0.42
i35				0.49					0.41	
i40			0.54							0.63

F_1_ represents “verbalizing,” F_2_ represents “fantasizing,” F_3_ represents “identifying,” F_4_ represents “emotionalizing,” and F_5_ represents “analyzing.”

Comparison of the factor loadings between the full sample and the analysis sample showed few notable differences. Deletion of the aberrant item-score patterns removed the cross loadings for items in the subscales verbalizing and fantasizing. In the complete sample, the identifying items 8, 18, 23, and 33 loaded on analyzing instead of identifying, but in the analysis sample, all items loaded on the postulated factors, with low cross loadings for items 13 and 28 on analyzing. Interestingly, these items are the counter-indicative items, and the results suggest that these items are indicators of analyzing rather than identifying. In the complete sample, the factor loadings showed an unsystematic pattern. In the analysis sample, the indicative items (10, 20, 30, and 40) loaded on the postulated factor but only item 40 had a substantial loading (>0.60), two items (25 and 35) had weak cross loadings on other factors, and the other items (5 and 15) loaded on none of the factors. Hence, the subscale analyzing could not be replicated in the complete and the analysis samples.

Table 3 shows the estimated factor correlations and the second-order factor structure based on the estimated factor correlations, in both the complete and the analysis sample. Results for verbalizing, fantasizing, identifying, and emotionalizing were consistent across different EFAs, corroborating the presence of a cognitive and an affective domain within the BVAQ. The affective dimension emotionalizing showed a substantive cross loading with the cognitive dimension in the complete sample but not in the analysis sample. Results for analyzing were ambiguous.

**Table 3:** Correlations between the first-order factors of the five-factor model and standardized second-order factor loadings in the analysis sample.

Subscales of the Bermond–Vorst Alexithymia Questionnaire	Estimated inter-factor correlations					Second-order factor loadings	
	
	Verbalizing	Fantasizing	Identifying	Emotionalizing	Analyzing	G1	G2
**All items (complete sample)**							
Verbalizing	1.00					0.83	
Fantasizing	0.16	1.00					0.85
Identifying	0.47	0.08	1.00			0.79	
Emotionalizing	0.25	0.31	0.14	1.00		0.34	0.76
Analyzing	0.52	0.31	0.49	0.46	1.00	0.82	0.47

**All items (analysis sample)**							
Verbalizing	1.00					0.51	
Fantasizing	0.21	1.00					0.62
Identifying	0.45	0.02	1.00			0.80	
Emotionalizing	0.33	0.39	0.15	1.00			0.66
Analyzing	0.23	0.32	0.11	0.36	1.00		0.53

**Indicative items (analysis sample)**							
Verbalizing	1.00					0.57	
Fantasizing	0.23	1.00					0.76
Identifying	0.45	0.09	1.00			0.73	
Emotionalizing	0.41	0.41	0.20	1.00			0.58
Analyzing	0.51	0.49	0.42	0.52	1.00	0.60	0.37

**Counter-indicative items (analysis sample)**							
Verbalizing	1.00					0.59	
Fantasizing	0.09	1.00					0.45
Identifying	0.39	−0.03	1.00			0.74	
Emotionalizing	0.12	0.31	0.05	1.00			0.70
Analyzing	0.48	0.20	0.50	0.41	1.00	0.75	0.29

*G1 represents the cognitive factor and G2 represents the affective factor*.

### Reliability

Table 4 (columns 3) shows coefficient alpha and corresponding 95% CIs for the first-order test scores, and the second-order test scores. Coefficient alpha ranged from 0.75 to 0.89. PRMSEs for the first-order test scores (column 4) were higher than the PRMSE for the second-order test scores or total scores (column 5). Table 4 (column 2) also shows the range of item-rest correlations of the items constituting the first-order test scores and the second-order test scores in the general population. Item-rest correlations suggested adequate assignment of the individual items to the subscales. These results also showed that some items are weak indicators of the general attribute of alexithymia. In particular, item 2 (*Before I fall asleep, I imagine all kinds of events, encounters and conversations*), item 5 (*I hardly ever consider my feelings*) and 15 (*When I feel uncomfortable, I will not trouble myself even more by asking myself why*) are weak indicators.

**Table 4:** Reliability and additional values (PRMSEs) of the first-order and second-order scores of the BVAQ (results obtained in the total sample).

Subscales	Range item–rest score correlations	Coefficient alpha (95% CI)	PRMSE	
			
			First-order scores	Total score
**First-order scores**				
Emotionalizing	0.31–0.55	0.75 (0.73–0.77)	0.75	0.53
Fantasizing	0.35–0.70	0.82 (0.80–0.84)	0.82	0.68
Identifying	0.39–0.55	0.79 (0.77–0.81)	0.79	0.65
Analyzing	0.39–0.60	0.80 (0.78–0.82)	0.80	0.68
Verbalizing	0.39–0.64	0.83 (0.81–0.85)	0.83	0.71

**Second-order scores**				
Cognitive dimension	0.30–0.63	0.89 (0.88–0.90)	0.89	0.74
Affective dimension	0.27–0.58	0.82 (0.80–0.84)	0.81	0.57

*95% CI, 95% confidence interval; PRMSE, Proportional Reduction in Mean Squared Error; BVAQ, Bermond–Vorst Alexithymia Questionnaire*.

*Range item–rest score correlations, coefficient alpha, and PRMSE were calculated for the general population (total sample)*.

### External Validity

**Figure 2:**
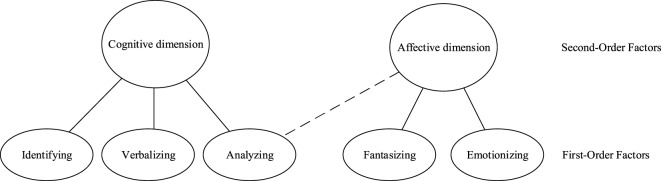
Second-order factor structure of the Bermond–Vorst Alexithymia Questionnaire based on exploratory factor analyses on the indicative items and on the counter-indicative items (analysis sample).

Table 5 shows results for EFAs for the indicative and the counter-indicative items. For both sets of items, the five-factor model fitted the data well and all items loaded on the corresponding factor. Cross loadings were absent. These results suggest that the items can be clustered into subscales as intended, but the counter-indicative items of analyzing may represent a slightly different conceptualization than the indicative items. Figure 2 shows a visualization of the factor structure for the indicative and the counter-indicative items.

**Table 5:** Standardized factor loadings of the five-factor model in the LISS panel data of the analysis sample, for the indicative items and counter-indicative items.

	Indicative items					Counter-indicative items				
	F1	F2	F3	F4	F5		F1	F2	F3	F4	F5
**Items from Vorst and Bermond’s subscale verbalizing**											

Items						Items					
i1	0.68					i6	0.63				
i11	0.69					i16	0.65				
i21	0.49					i26	0.64				
i36	0.48					i31	0.66				

**Items from Vorst and Bermond’s subscale fantasizing**											

Items						Items					
i7		0.74				i2		0.45			
i17		0.53				i12		0.77			
i22		0.92				i27		0.77			
i32		0.65				i37		0.55			

**Items from Vorst and Bermond’s subscale identifying**											

Items						Items					
i8			0.60			i3			0.60		
i18			0.60			i13			0.49		
i23			0.65			i28			0.79		
i33			0.69			i38			0.59		

**Items from Vorst and Bermond’s subscale emotionalizing**											

Items						Items					
i4				0.50		i14				0.59	
i9				0.63		i19				0.53	
i24				0.50		i29				0.75	
i34				0.59		i39				0.69	

**Items from Vorst and Bermond’s subscale analyzing**											

Items						Items					
i5					0.35	i10					0.51
i15					0.39	i20					0.68
i25					0.59	i30					0.52
i35					0.60	i40					0.80

*Only loadings of 0.3 or higher are reported*.

F_1_ represents “verbalizing,” F_2_ represents “fantasizing,” F_3_ represents “identifying,” F_4_ represents “emotionalizing,” and F_5_ represents “analyzing.”

Table 3 also shows estimated factor correlations and the second-order factor structure based on the estimated factor correlations for indicative and counter-indicative items. Correlations of analyzing with the other factors were lower when analyzing all 40 items together than for indicative and counter-indicative items separately. The different factor correlations for indicative and counter-indicative items might also explain the differences between the second-order factor structures. Results suggest that indicative and counter-indicative analyzing items refer to slightly different attributes, which is obscured when analyzing all items together.

### Scoring

Table 6 (columns 2–5) shows the means and SDs of the first-order test scores and the second-order test scores for the SSRD sample and for the general-population sample. Table 6 (columns 6–7) also shows the *p*-values and Cohen’s *d* for the comparison between the SSRD sample and the general-population sample. The mean scores of emotionalizing (*p* < 0.001, *d* = 0.57) and the affective dimension (*p* = 0.003, *d* = 0.22) were significantly higher in the general-population sample. The mean scores on identifying (*p* < 0.001, *d* = −0.57), verbalizing (*p* < 0.001, *d* = −0.35), and the cognitive dimension (*p* < 0.001, *d* = −0.33) were significantly lower in the general-population sample than the SSRD sample.

**Table 6:** Descriptive statistics, of the first-order and second-order scores of the BVAQ (results obtained in the total sample), descriptive statistics for the SSRD sample (*N* = 234) and statistical comparison between SSRD sample and general population of BVAQ scores.

Subscales	Descriptive statistics (SSRD sample)		Descriptive statistics (general population)		BVAQ scores comparison	
	M	SD	M	SD	*p*	*d*
**First-order scores**						
Emotionalizing	18.8	5.2	21.7	5.1	<0.001	0.57
Fantasizing	26.8	6.9	25.8	6.4	0.056	−0.15
Identifying	22.1	7.0	18.9	5.2	<0.001	−0.57
Analyzing	19.6	6.0	20.4	5.3	0.065	0.15
Verbalizing	25.4	8.3	23.1	6.0	<0.001	−0.35

**Second-order scores**						
Cognitive dimension	67.1	17.6	62.3	13.4	<0.001	−0.33
Affective dimension	45.5	8.9	47.5	9.3	0.003	0.22

*SSRD, Somatic symptom and related disorder; BVAQ, Bermond–Vorst Alexithymia Questionnaire*.

Inspection of the residuals suggested that BVAQ total scores were linearly related to age and that heteroscedasticity was absent. Table 7 (columns 2–4) shows the estimated unstandardized regression coefficients for predicting first-order test scores and second-order test scores by age and gender. Age and gender explained 2% (identifying) to 15% (emotionalizing) of the variance of the first-order test scores (Table 7, column 5), which amounts to small to medium effects according to Cohen’s (59) rules of thumb. Except for fantasizing, a significant effect of gender was found for the other subscales. Significant effects of age were found for the subscales fantasizing, analyzing, and the affective factor. To gauge the practical importance of age given the estimated regression model, we looked at differences between predicted scores for the youngest and the oldest respondents. The predicted score of 18-year-old males equaled 46.9, whereas the predicted score for an 89-year-old male equaled 53.3, which represents a score difference of 6.4 units. Based on the distribution of the residuals (i.e., SD = 8.84; see Table 7), a score difference of 6.4 units amounts to Cohen’s *d* of 0.73 (6.4/8.84), meaning a large effect size. Therefore, it is important to control for age.

**Table 7:** Multiple regression analysis predicting first-order scores or second-order scores from age and gender, and distribution of the residuals.

Multiple regression analysis					Distribution of residuals		
Bermond–Vorst Alexithymia questionnaire score	Constant	Regression effect (*B*)^a^		*R*-square	SD	Kurtosis	Skewness
			
			Gender^b^	Age				
Emotionalizing	24.07 (0.50)	−**4.00** (0.30)	−0.01 (0.01)	0.15	4.66	−0.12	0.07
Fantasizing	21.20 (0.67)	−0.63 (0.40)	**0.10** (0.01)	0.08	6.17	−0.22	−0.16
Identifying	18.93 (0.55)	−**1.36** (0.33)	0.01 (0.01)	0.02	5.12	−0.14	0.04
Analyzing	19.89 (0.55)	−**2.33** (0.33)	**0.03** (0.01)	0.07	5.08	0.12	0.02
Verbalizing	24.67 (0.63)	−**2.59** (0.38)	0.00 (0.01)	0.05	5.81	−0.32	0.10
Cognitive dimension	63.50 (1.40)	−**6.28** (0.84)	0.04 (0.02)	0.06	12.94	0.14	−0.14
Affective dimension	45.27 (0.96)	−**4.63** (0.57)	**0.09** (0.02)	0.10	8.84	0.07	−0.09

*^a^Unstandardized partial regression coefficients (SE in parentheses)*.

*^b^Reference category is men*.

*Estimated regression coefficients printed in boldface are significant at the 1% level*.

Table 7 (columns 6–8) also describes the distribution of the residuals (i.e., SD, skewness, and kurtosis). In all models, residuals were obtained for the model including both age and gender as predictors. The residuals were normally distributed. The coefficients in Table 7 can be used to norm scores that take age and gender differences into account. An Excel template for this purpose is available upon request from the corresponding author as well as norm tables for each age and gender group.

## Discussion

This study was the first to validate the BVAQ for the general population. Aberrant item responses due to extreme responders were removed prior to the EFA in an effort to better validate the BVAQ factor structure. Removal of aberrant item-response patterns produced a factor structure that was consistent with the conceptualization of alexithymia. This study showed that person-fit analysis may contribute to a better understanding of the factor structure.

The results suggest that items indicative of analyzing represent a conceptually different attribute than counter-indicative items. A competing explanation for different results might be the wording of the items. For example, indicative items are phrased in terms of “unclear” whereas counter-indicative items are phrased in terms of “understand.” Such small differences may invoke different cognitive processes, producing responses that represent different attributes. Because this was the first study in the general population, it is unclear whether such wording effects are typical of the general population or whether these results also generalize to other populations. This is a topic for future research. Because the results showed a clear difference with respect to the second-order factor structure for the indicative and the counter-indicative items, and because analyzing ability also loaded on the affective factor instead of only on the cognitive factor, our analysis of indicative and counter-indicative items may explain why construct validity of the BVAQ was found suboptimal in earlier studies.

We found that the BVAQ is a reliable instrument. Additional analyses showed that when scores are aggregated to second-order test scores, reliable information about the constituent components is lost. Consequently, this study provided support for the use of first-order test scores to provide diagnostic information for understanding alexithymia at a more detailed level. Because first-order test scores have additional value with respect to second-order test scores, clinicians and researchers should better rely on the first-order test scores for a clinical judgment.

This was also the first study that compared alexithymia scores in the general population and in a patient population suffering from SSRD, that, we hypothesized, would have more difficulty expressing their feelings and thoughts about their symptoms. Consequently, they were expected to score higher on an alexithymia scale than the general population. Another possibility was that patients gave a more involved opinion about their symptoms because the data were collected in connection with their intake for treatment. We checked the likelihood of these alternative explanations. Because higher scores of alexithymia were found in the SSRD group, support for construct validity was found.

Regression analyses of alexithymia on age and gender corroborated the trends found in other studies. Males had higher mean alexithymia scores than women and a positive effect of age was found, similar to findings in studies using clinical populations [e.g., Salminen et al. (37), Franz et al. (60), Joukamaa et al. (61), Mattila et al. (62), Pasini et al. (63)]. Caution should be exercised drawing conclusions about within-person change in alexithymia over time based on cross-sectional data. Individuals in varying cohorts may grow up in different social contexts, which may produce between-person variation in mean alexithymia across age groups, while alexithymia remains stable within persons. Longitudinal data are needed to study within- and between-person differences in alexithymia over time while controlling for physical conditions. This is a topic for future research.

Normative data were reported, both unconditional and conditional on age and gender. Both types of norms have practical value, but should be used carefully. When using age and gender-specific norms, one implicitly assumes that gender and age differences in alexithymia are related to contextual factors and not the construct itself. Contextual factors may include social environment and time-specific social norms. For example, two persons with the same BVAQ scores but of different age may not be conceived as equally alexithymic because the older person grew up in times where it was socially not that well accepted to talk about emotions while the younger person is more used to it. Likewise, a male and female having the same BVAQ scores may not be equally alexithymic because the female may have learned to express her emotions when she was young while the male did not. Hence, gender differences result from social norms and not the trait itself and this effect should be partialed out when comparing BVAQ scores between males and females. However, in the clinical practice, where the BVAQ is used for screening and treatment decisions, one may not want to treat males and females with the same BVAQ total scores differently. In such cases, clinicians can use the unconditional scores. We may notice that screening using unconditional norms may result in different prevalence rates for males and females or across age cohorts, while prevalence rates will be the same when using conditional norms.

Previous studies showed a relationship between alexithymia and distress (64–69). Distress can be an outcome or a determinant of alexithymia (70), but this topic did not receive much attention yet. Tominaga et al. (71) suggested that alexithymia hampers the successful regulation of negative affect and leads to increased distress. Distress also has been shown to coincide with alexithymia as a state-dependent phenomenon (72, 73). Because the role of distress for alexithymia is unclear, future studies may address this topic.

Significant differences were found between responders and non-responders with respect to age, educational level, and marital status. Because age is associated with alexithymia, caution should be exercised when generalizing results to the general population. Another limitation involves the use of panel data. However, we corrected for extreme responders to mitigate this limitation. The BVAQ is more reliable than the TAS-20, which is possibly due to the former questionnaire’s greater number of items.

The development of tools to assess alexithymia is continuing. The TSIA (21) enables the measurement of fantasizing that was lacking in the TAS-20 and is considered important (19, 20), Also, a recent study reported that the subscale external oriented thinking of the TAS-20 has weak psychometric properties in the group of younger adolescents (74). Another study corroborated this finding (75) and concluded that the psychometric properties of the external oriented thinking subscale are poorer than those of identifying and describing feelings. Based on the findings of this study, the BVAQ could be a reliable alternative to the TAS-20 that is based on a different operationalization of the alexithymia construct.

As far as we know, this was the first study exploring the BVAQ factor structure in the general population, taking external validity into account, and comparing the general population with a patient population expected to score higher on alexithymia. The currently existing treatment options for alexithymia are not effective and the development of evidence-based treatments is necessary (76). The psychotherapeutic process relies primarily on the ability of the patient to access their emotions. Patients who are unable to do so are difficult to treat by the therapist. In order to develop evidence-based treatments, a proper assessment of alexithymia is pivotal. Hence, the results of our study provide clinicians with a valuable tool for assessing alexithymia in the clinic by using the norm scores and offer clinicians and scientists a starting point for the development of evidence-based treatment options. This study thus provided insight in the reliability of the BVAQ and provided norm scores for use in clinical practice in the Netherlands.

## Ethics Statement

The Commission of Scientific Research of GGz Breburg approved to conduct this study (file number: CWO 2014-09). Patients gave consent to make use of their intake data for scientific research purposes.

## Author Contributions

LV drafted the manuscript. LV and WE were responsible for design and analysis of the data. WE, KS, and CvdF-C revised the draft. All authors approved of the final manuscript.

## Conflict of Interest Statement

The authors declare that the research was conducted in the absence of any commercial or financial relationships that could be construed as potential conflict of interest.
